# Supplementary material for: FastqPuri: high-performance preprocessing of RNA-seq data
Source: BMC Bioinformatics. 2019 May 3;20:226. doi: 10.1186/s12859-019-2799-0 (PMC6500068; doi:10.1186/s12859-019-2799-0)
Supplement: Supplementary file 2 — Archive of FastqPuri. Archive containing all files needed to install and run FastqPuri v1.0.6. Date stamp March 22, 2019. (GZ 47,819 kb) [file 12859_2019_2799_MOESM2_ESM.gz › FastqPuri-1.0.6/html/io__trimFilter_8c.html]

FastqPuri: src/io\_trimFilter.c File Reference


FastqPuri


- src

Functions

io\_trimFilter.c File Reference

buffer fq output, write summary file
More...

`#include <stdio.h>`  
`#include <stdlib.h>`  
`#include <string.h>`  
`#include "io_trimFilter.h"`  
`#include "defines.h"`

Include dependency graph for io\_trimFilter.c:

|  |  |
| --- | --- |
| Functions | |
| void | buffer\_output (FILE \*fout, const char \*str, const int len, const int fd\_i) |
|  | buffers the output before writing to disk, writes out summary More... |
|  | |
| void | write\_summary\_TF (Stats\_TF tf\_stats, char \*filename) |
|  | writes stats of filtering to summary file (binary) |
|  | |

## Detailed Description

buffer fq output, write summary file

Author
:   Paula Perez paula.nosp@m.pere.nosp@m.zrubi.nosp@m.o@gm.nosp@m.ail.c.nosp@m.om

Date
:   29.08.2017

## Function Documentation

## ◆ buffer\_output()

|  |  |  |  |
| --- | --- | --- | --- |
| void buffer\_output | ( | FILE \* | *fout*, |
|  |  | const char \* | *str*, |
|  |  | const int | *len*, |
|  |  | const int | *fd\_i* |
|  | ) |  |  |

buffers the output before writing to disk, writes out summary

Parameters
:   |  |  |
    | --- | --- |
    | fout | FILE pointer where we might write to disk; |
    | str | string we want to add |
    | len | length of the string we want to add |
    | fd\_i | identifier: GOOD, ADAP, CONT, LOWQ, NNNN |


---

Generated on Mon Mar 19 2018 23:42:01 for FastqPuri by  

 1.8.14
